# Nurse-performed diaphragm ultrasound integrated with spontaneous breathing trial criteria for risk stratification of extubation outcomes in neurosurgical critically ill patients: a multicenter prospective cohort study

**DOI:** 10.3389/fmed.2026.1732944

**Published:** 2026-02-09

**Authors:** Yifan Jiang, Bingxin Wang, Hai Wang, Jingyi Wang, Jianwen Xu, Yuli Liu, Li Wang, Yajuan Zhang, Fang Fang, Kaihong Fei

**Affiliations:** 1 Shanghai General Hospital, Shanghai, China; 2 Shanghai Chest Hospital, Shanghai Jiao Tong University School of Medicine, Shanghai, China; 3 Yangpu District Shidong Hospital of Shanghai, Shanghai, China; 4 Shanghai Yangzhi Rehabilitation Hospital, Shanghai, China; 5 Shanghai Tenth People's Hospital, Shanghai, China; 6 Shanghai Sixth People’s Hospital Affiliated to Shanghai Jiao Tong University School of Medicine, Shanghai, China

**Keywords:** diaphragm ultrasound, mechanical ventilation weaning, neurosurgical intensive care unit, reintubation, spontaneous breathing trial

## Abstract

**Background:**

In neurosurgical intensive care units (ICUs), conventional spontaneous breathing trial (SBT) criteria may not adequately reflect diaphragm function, which can contribute to premature or delayed extubation. Diaphragm ultrasound provides objective bedside measures to support individualized weaning.

**Methods:**

This multicenter prospective cohort study enrolled 188 patients from six tertiary hospitals. Patients were sequentially managed in three phases: control (conventional SBT), diaphragm thickening fraction (DTF)–guided management (extended SBT if DTF was below the prespecified threshold), and combined DTF plus diaphragmatic excursion (DE)–guided management. Primary outcomes included first SBT success, first extubation success, reintubation, and duration of mechanical ventilation. Receiver operating characteristic (ROC) and Cohen’s kappa analyses were used to evaluate predictive accuracy and agreement between SBT results and extubation outcomes.

**Results:**

No significant differences were observed in first SBT success or first extubation success (*p* = 0.127; *p* = 0.900). Reintubation occurred significantly more often in the control group than in the DTF and DTF + DE groups (*p* = 0.004). Total ventilation time was also longer in the control group (*p* < 0.001). ROC analysis demonstrated stronger predictive value for DTF than for DE, with limited incremental benefit from combining the two measures (*p* < 0.01). Kappa analysis showed improved agreement between SBT results and extubation outcomes when diaphragm-based stratification was applied (*p* < 0.01).

**Conclusion:**

Nurse-performed diaphragm ultrasound can be implemented to support physician-led weaning assessment. DTF-based stratification was associated with reduced reintubation and shorter ventilation duration.

## 1 Introduction

Mechanical ventilation is a critical life-support measure for neurosurgical critically ill patients, yet prolonged ventilation increases the risk of ventilator-associated complications, delayed extubation, and mortality (1). Therefore, balancing timely liberation from mechanical ventilation with patient safety remains a central clinical challenge in neurocritical care (2). Unlike general intensive care units (ICUs), the weaning process in neurosurgical ICUs is predominantly nurse-led: due to rapid fluctuations in neurological status and respiratory dynamics, nurses perform assessments of consciousness, circulation, and respiratory readiness after sedation interruption and initiate the spontaneous breathing trial (SBT), and their judgment directly influences the timing of extubation (3).

However, conventional SBT criteria rely primarily on short-term respiratory and physiological responses and therefore fail to capture the endurance and functional reserve of the diaphragm (4). Some patients are able to tolerate a 30 min SBT yet experience extubation failure due to respiratory muscle fatigue afterward; conversely, patients who actually possess adequate weaning capacity may show transient instability during more demanding SBT conditions, leading to unnecessary delays in extubation (5). These issues highlight the lack of a quantitative, bedside-accessible indicator of diaphragmatic function that can support stratified decision-making in current clinical practice.

In recent years, diaphragm ultrasound has been increasingly utilized owing to its noninvasive, real-time, and repeatable nature, enabling the assessment of diaphragm thickening fraction (DTF) and diaphragm excursion (DE). Numerous studies have demonstrated its predictive value for extubation outcomes (6, 7). However, most existing evidence is observational, single-center, or performed primarily by physicians, and there is a lack of prospective studies evaluating how nurse-led diaphragm assessment can guide the selection of SBT strategies in neurosurgical ICUs. In particular, evidence is insufficient regarding whether diaphragm-based stratification can reduce reintubation, improve the consistency between SBT performance and actual weaning outcomes, or shorten the duration of mechanical ventilation—key clinical endpoints in neurocritical care.

To date, however, evidence remains limited regarding how diaphragm ultrasound can be integrated into nurse-led workflows to guide the selection of SBT strategies in neurosurgical ICUs (8). Therefore, this multicenter prospective study was conducted to determine whether incorporating diaphragm ultrasound into routine nursing assessment could provide a more physiologically informed, individualized approach to weaning in neurosurgical critically ill patients.

## 2 Methods

### 2.1 Study design and participants

This was a multicenter, prospective cohort study. Participants were neurosurgical critically ill patients admitted between May 2024 and April 2025 to the general ICUs of six tertiary hospitals in Shanghai. All six hospitals were regional medical centers, ensuring representativeness and heterogeneity of the study population. Each center independently recruited patients using convenience sampling, with screening based on unified inclusion and exclusion criteria.

Inclusion criteria were: (1) admission to ICU with GCS < 12; and (2) age ≥18 years. Exclusion criteria were: (1) factors such as trauma fixation or skin conditions affecting diaphragmatic motion or ultrasound assessment; (2) invasive or noninvasive ventilation before admission; and (3) pregnancy or lactation. Elimination criteria were: (1) duration of invasive ventilation <24 h during the study; and (2) patient or family request to withdraw from the trial.

Patients were allocated sequentially according to admission time into three phases with distinct weaning management processes:

1. Control period, May–August 2024: conventional weaning (control group).
2. DTF period, September–December 2024: DTF-based weaning (DTF group).
3. DTF + DE period, January–April 2025: combined DTF and DE weaning (DTF + DE group).

Sequential allocation by time avoided overlap of multiple processes in the same period and ensured integrity and feasibility of each intervention. All centers followed a unified protocol for patient screening, data collection, and process execution to guarantee cross-center comparability and consistency.

### 2.2 Study methods

#### 2.2.1 Interventions and procedures

In this study, a SBT was defined as ~30 min of breathing under low-level pressure support ventilation (PSV) (PS 8 cmH₂O, PEEP 5 cmH₂O, FiO₂ ≤ 40%). For high-risk patients identified by diaphragm ultrasound thresholds, we applied a protocolized extended observation window (up to 6 h) under the same minimal PSV support as a bedside safety verification step to detect delayed intolerance prior to physician-led extubation decisions. The study consisted of three consecutive phases, each applying one fixed weaning management process.

Common procedures (applied to all phases): Sedation was interrupted daily at 05:30 by the responsible nurse. When the Richmond Agitation–Sedation Scale (RASS) score reached ≥ − 2, patients were evaluated against conventional readiness-to-wean criteria, including respiratory, circulatory, oxygenation, and consciousness status. Criteria included: respiratory rate ≤35/min; adequate oxygenation defined as SpO₂ ≥ 90%, FiO₂ ≤ 40%, PEEP ≤8 cmH₂O, or PaO₂/FiO₂ ≥ 150 with PEEP ≤8 cmH₂O; effective cough reflex with secretion clearance; RASS −2 to +1; no continuous sedatives; and hemodynamic stability with none or minimal vasopressors. Eligible patients underwent bedside measurement of diaphragm thickening fraction (DTF) and diaphragmatic excursion (DE) under low-level PSV before SBT. During the standard SBT and, when applicable, throughout the extended observation window, patients were continuously monitored for respiratory, circulatory, and oxygenation indices. Failure criteria were predefined as: respiratory rate >35/min; SpO₂ < 90% uncorrected by FiO₂/flow increase; heart rate >140/min or systolic blood pressure <90 or >180 mmHg with signs of hypoperfusion; or decreased consciousness, anxiety, or agitation. If failure criteria were met, ventilatory support was resumed first, and sedation was then restarted as clinically indicated; reassessment was performed on the following day. After extubation, continuous sedation was not routinely resumed; sedatives and/or analgesics were re-initiated only after reassessment when clinically indicated (e.g., agitation or pain). Nurses documented diaphragm parameters and all SBT/observation outcomes, which were reviewed during morning rounds for physician-led extubation decisions.

Baseline physiological variables reported as “before SBT” (mean arterial pressure, PEEP, tidal volume, heart rate, and body temperature) were recorded immediately prior to SBT initiation at the time of the pre-SBT assessment under low-level PSV, using values displayed on the bedside monitor and ventilator.

#### 2.2.2 Phase-specific decision rules

1. Control phase (conventional SBT process): Patients meeting readiness-to-wean criteria proceeded with a standard ~30 min SBT under low-level PSV. Patients tolerating 30 min with stable indices were deemed SBT success.
2. DTF phase (DTF-based process): Patients meeting readiness-to-wean criteria underwent pre-SBT diaphragm ultrasound as above. If DTF ≥ 20%, the patient proceeded with a standard ~30 min SBT under low-level PSV. If DTF < 20%, the patient entered an extended observation window (up to 6 h) under the same PSV settings as a safety verification step to detect delayed intolerance. This period was not a separate SBT, but a protocolized continuation of minimal support with the same predefined failure criteria and continuous bedside monitoring. Patients maintaining respiratory rate ≤35/min, SpO₂ ≥ 90%, and stable hemodynamics throughout the observation window were considered eligible for extubation; otherwise, ventilatory support was resumed with reassessment on the following day.
3. DTF+DE phase (combined DTF and DE process): Patients meeting readiness-to-wean criteria underwent pre-SBT diaphragm ultrasound as above. If DTF ≥ 20% and DE ≥ 1.0 cm, the patient proceeded with a standard ~30 min SBT under low-level PSV. If either parameter was below threshold, the patient entered an extended observation window (up to 6 h) under the same PSV settings as a safety verification step to detect delayed intolerance. This period was not a separate SBT, but a protocolized continuation of minimal support with the same predefined failure criteria and continuous bedside monitoring. Patients maintaining respiratory rate ≤35/min, SpO₂ ≥ 90%, and stable hemodynamics throughout the observation window were considered eligible for extubation; otherwise, ventilatory support was resumed with reassessment on the following day.

#### 2.2.3 Diaphragm ultrasound assessment

In all patients, once daily sedation was stopped and SBT eligibility was confirmed, the responsible nurse performed diaphragm ultrasound assessment prior to SBT initiation. Patients were positioned semi-recumbent with the head of bed elevated ~30°. Under low-level PSV (PS 8 cmH₂O, FiO₂ ≤ 40%, PEEP 5 cmH₂O), evaluations included DE, end-inspiratory thickness, end-expiratory thickness, and calculation of DTF. Ultrasound was conducted using a LOGIQ E device (GE Healthcare, United States), performed by ICU nurses certified in critical care ultrasound at each center.

1. DE assessment: Patients were placed supine with the head of bed elevated 30°. A 3.5–5 MHz convex probe was positioned at the intersection of the right midclavicular or anterior axillary line and the costal margin, using the liver as an acoustic window. The probe was directed cranially and dorsally, aligning the sampling line perpendicular to the diaphragm. Using M-mode, diaphragmatic motion amplitude was measured by recording distances at end-inspiration and end-expiration relative to baseline. Three consecutive measurements were averaged. DE (mm) = inspiratory distance from baseline − expiratory distance from baseline.
2. DTF assessment: Patients were positioned supine with the head of bed elevated 30°. A 7–12 MHz linear probe was placed in the right midaxillary line at the 8th–10th intercostal space, using the liver as an acoustic window. Diaphragm thickness variation was observed in M-mode, recording DTe and DTi. For each patient, 3–5 respiratory cycles were measured and averaged. DTF (%) = (DTi − DTe) / DTe × 100%.

### 2.3 Outcome measures

1. First weaning success rate: defined as the proportion of patients who, after their first SBT and extubation, did not require reintubation, invasive or noninvasive ventilation, and did not die within 48 h, used to evaluate the direct impact of optimized processes on weaning success.
2. First SBT success rate: defined as the proportion of patients who tolerated the entire first 30 min SBT with stable physiological indices and met extubation criteria, used to assess the impact of diaphragm ultrasound screening on the accuracy of SBT evaluation.
3. Reintubation rate: defined as the proportion of patients who required invasive or noninvasive ventilation within 48 h after extubation due to respiratory failure, secretion retention, or other causes.
4. Consistency between first SBT and first weaning outcome: defined as agreement between first SBT evaluation and actual weaning outcome (successful or failed extubation), used to analyze the diagnostic accuracy of diaphragm ultrasound combined with SBT criteria in weaning decision-making.
5. Mechanical ventilation duration: defined as the cumulative invasive ventilation time (hours) from initiation of the first SBT until final successful weaning, used to evaluate the impact of optimized processes on ventilator dependence.
6. 14-day mortality: defined as the proportion of patients who died within 14 days from initiation of the first SBT, used to evaluate the impact of optimized weaning on short-term prognosis.

### 2.4 Operator training and measurement quality control

To minimize operator-dependent variability across centers, diaphragm ultrasound was performed by ICU nurses certified in critical care ultrasound at each site. Prior to study initiation, all operators completed standardized training on probe positioning, image acquisition, and measurement definitions for DTF and DE, using a unified operating manual with step-by-step instructions. During data collection, measurements were obtained under the same ventilator settings and patient position (semi-recumbent, head of bed ~30°). For each parameter, multiple consecutive breaths were recorded and averaged (DE: three measurements; DTF: 3–5 respiratory cycles), and images were required to meet prespecified quality criteria (clear diaphragm interface and stable M-mode tracing). If image quality was inadequate or measurements were inconsistent across breaths, acquisition was repeated. Each center conducted routine internal checks of stored images and measurements by a designated senior ultrasound-trained nurse to ensure protocol adherence.

### 2.5 Statistical analysis

All data were analyzed using SPSS 26.0. Continuous variables were tested for normality. Normally distributed data were expressed as mean ± SD and compared among groups using one-way ANOVA; pairwise comparisons were conducted with LSD (homogeneity of variance) or Tamhane’s T2 (heterogeneity). Non-normally distributed data were expressed as median (IQR) and compared among groups using the Kruskal–Wallis test. Categorical variables were presented as *n* (%) and compared among groups using chi-square or Fisher’s exact test. All tests were two-sided with significance set at *p* < 0.05.

Group comparisons of first weaning success were performed using chi-square tests. ROC curves were plotted to evaluate the predictive value of diaphragm ultrasound parameters for first weaning and first SBT success, with AUC calculated. Consistency between first SBT and first weaning outcomes was analyzed using Kappa statistics, with significance set at *p* < 0.05.

### 2.6 Ethical approval

This study was approved by the Ethics Committee of Shanghai General Hospital (Approval No. YL-2025-078) and by the institutional review boards of all participating centers. The study was conducted in accordance with the Declaration of Helsinki. Written informed consent was obtained from the patients or their legal representatives prior to enrollment.

## 3 Results

### 3.1 Baseline characteristics and diaphragm function during SBT

There were no statistically significant differences among the three groups in demographic and clinical baseline characteristics, including age (*p* = 0.572), sex (*p* = 0.567), and primary diagnosis distribution (*p* = 0.945). Before SBT, mean arterial pressure (*p* = 0.989), PEEP (*p* = 0.109), tidal volume (*p* = 0.388), heart rate (*p* = 0.331), and body temperature (*p* = 0.918) did not differ significantly among the groups. Regarding diaphragm function assessed before SBT, end-inspiratory diaphragm thickness (*p* = 0.815), end-expiratory diaphragm thickness (*p* = 0.692), diaphragm thickening fraction (*p* = 0.386), and diaphragmatic excursion (*p* = 0.082) were also comparable across the three groups (Table 1).

**Table 1:** Comparison of baseline characteristics and diaphragm function data during SBT among groups.

Variable	Category	Control (*n* = 62)	DTF (*n* = 65)	DTF + DE (*n* = 61)	*F/*χ^2^	*P*
Age (years)		60.19 ± 17.48	57.73 ± 16.28	60.67 ± 16.63	0.561	0.572
Sex	Male (*n*, %)	40 (64.52)	37 (56.92)	39 (63.93)	0.965	0.617
	Female (*n*, %)	22 (35.48)	28 (43.08)	22 (36.07)		
Primary diagnosis	TBI (*n*, %)	23 (37.10)	17 (26.15)	18 (29.51)	2.842	0.944
	Cerebral infarction (*n*, %)	5 (8.06)	5 (7.69)	4 (6.56)		
	Intracerebral hemorrhage (*n*, %)	26 (41.94)	30 (46.15)	28 (45.90)		
	Brain tumor (*n*, %)	3 (4.84)	6 (9.23)	4 (6.56)		
	Others (*n*, %)	5 (8.06)	7 (10.77)	7 (11.48)		
Baseline data before SBT	Mean arterial pressure (mmHg)	85.64 ± 10.92	85.96 ± 14.38	85.72 ± 12.21	0.011	0.989
	PEEP (cmH_2_O)	7.09 ± 3.39	6.91 ± 4.06	5.79 ± 3.57	2.244	0.109
	Tidal volume (mL)	524.96 ± 125.05	552.55 ± 110.81	538.88 ± 100.79	0.951	0.388
	Heart rate (beats/min)	97.19 ± 8.08	99.01 ± 8.17	99.29 ± 9.29	1.111	0.331
	Temperature (°C)	36.46 ± 0.65	36.46 ± 0.61	36.42 ± 0.60	0.086	0.918
Diaphragm-related data before SBT	End-inspiratory diaphragm thickness (cm)	0.26 ± 0.10	0.25 ± 0.12	0.26 ± 0.08	0.205	0.815
	End-expiratory diaphragm thickness (cm)	0.20 ± 0.07	0.20 ± 0.09	0.21 ± 0.06	0.369	0.692
	Thickening fraction (%)	24.30 ± 12.67	23.94 ± 12.31	26.85 ± 13.33	0.958	0.386
	Excursion (cm)	1.21 ± 0.47	1.04 ± 0.52	1.20 ± 0.44	2.534	0.082

Values are mean ± SD or *n* (%). SBT, spontaneous breathing trial; DTF, diaphragm thickening fraction; DE, diaphragmatic excursion; MAP, mean arterial pressure; PEEP, positive end-expiratory pressure; PSV, pressure support ventilation; FiO₂, fraction of inspired oxygen; TBI, traumatic brain injury. “Before SBT” variables were recorded immediately prior to SBT initiation during the pre-SBT assessment under low-level PSV. *P* values were derived from one-way ANOVA (continuous variables) or χ^2^ tests (categorical variables); F/χ^2^ indicates the corresponding test statistic.

### 3.2 Primary outcomes within 48 h and 14 days after SBT

Within 48 h after SBT, there was no significant difference among the three groups in the first SBT success rate (χ^2^ = 4.133, *p* = 0.127) or the first extubation success rate (χ^2^ = 0.211, *p* = 0.900). However, the incidence of reintubation differed significantly, with lower rates in the DTF and DTF + DE groups compared with the control group (χ^2^ = 10.981, *p* = 0.004). Short-term survival within 48 h showed no significant difference across groups (χ^2^ = 0.151, *p* = 0.927). Mechanical ventilation (MV) duration within 48 h was significantly shorter in the DTF and DTF + DE groups compared with controls (*F* = 8.509, *p* < 0.001).

At 14 days after SBT, survival remained comparable among the three groups (χ^2^ = 0.151, *p* = 0.927). By contrast, the total MV duration differed significantly, with markedly shorter times observed in the DTF and DTF + DE groups than in the control group (*F* = 9.025, *p* < 0.001) (Table 2).

**Table 2:** Analysis of primary outcomes within 48 hours and 14 days after SBT among groups.

Outcomes within 48 h after SBT						Outcomes within 14 days after SBT					
Outcome variables	Control (*n* = 62)	DTF (*n* = 65)	DTF + DE (*n* = 61)	*F/*χ^2^	*P*	Outcome variables	Control (*n* = 62)	DTF (*n* = 65)	DTF + DE (*n* = 61)	*F/*χ^2^	*P*
First SBT success (*n*, %)	50 (80.65)	42 (64.62)	41 (67.21)	4.133	0.127	Survival (*n*, %)	15 (24.19)	14 (21.54)	15 (24.59)	0.151	0.927
First extubation success (*n*, %)	41 (66.13)	40 (61.54)	40 (65.57)	0.211	0.900	MV duration (*d*)	2.82 ± 4.58	0.68 ± 2.11	0.80 ± 2.14	9.025	<0.001
Reintubation (*n*, %)	16 (25.81)	5 (7.69)	5 (8.20)	10.981	0.004						
Survival (*n*, %)	47 (75.81)	50 (76.92)	46 (75.41)	0.151	0.927						
MV duration (*d*)	0.61 ± 0.78	0.24 ± 0.40	0.26 ± 0.43	8.509	<0.001						

Values are mean ± SD or *n* (%). SBT, spontaneous breathing trial; DTF, diaphragm thickening fraction; DE, diaphragmatic excursion; MV, mechanical ventilation. Outcomes “within 48 h after SBT” were assessed from SBT initiation through 48 h; outcomes “within 14 days after SBT” were assessed from SBT initiation through day 14. *P* values were derived from one-way ANOVA (continuous variables) or χ^2^ tests (categorical variables); F/χ^2^ indicates the corresponding test statistic.

### 3.3 Predictive value of nurse-performed diaphragm ultrasound parameters for weaning failure

**Figure 1:**
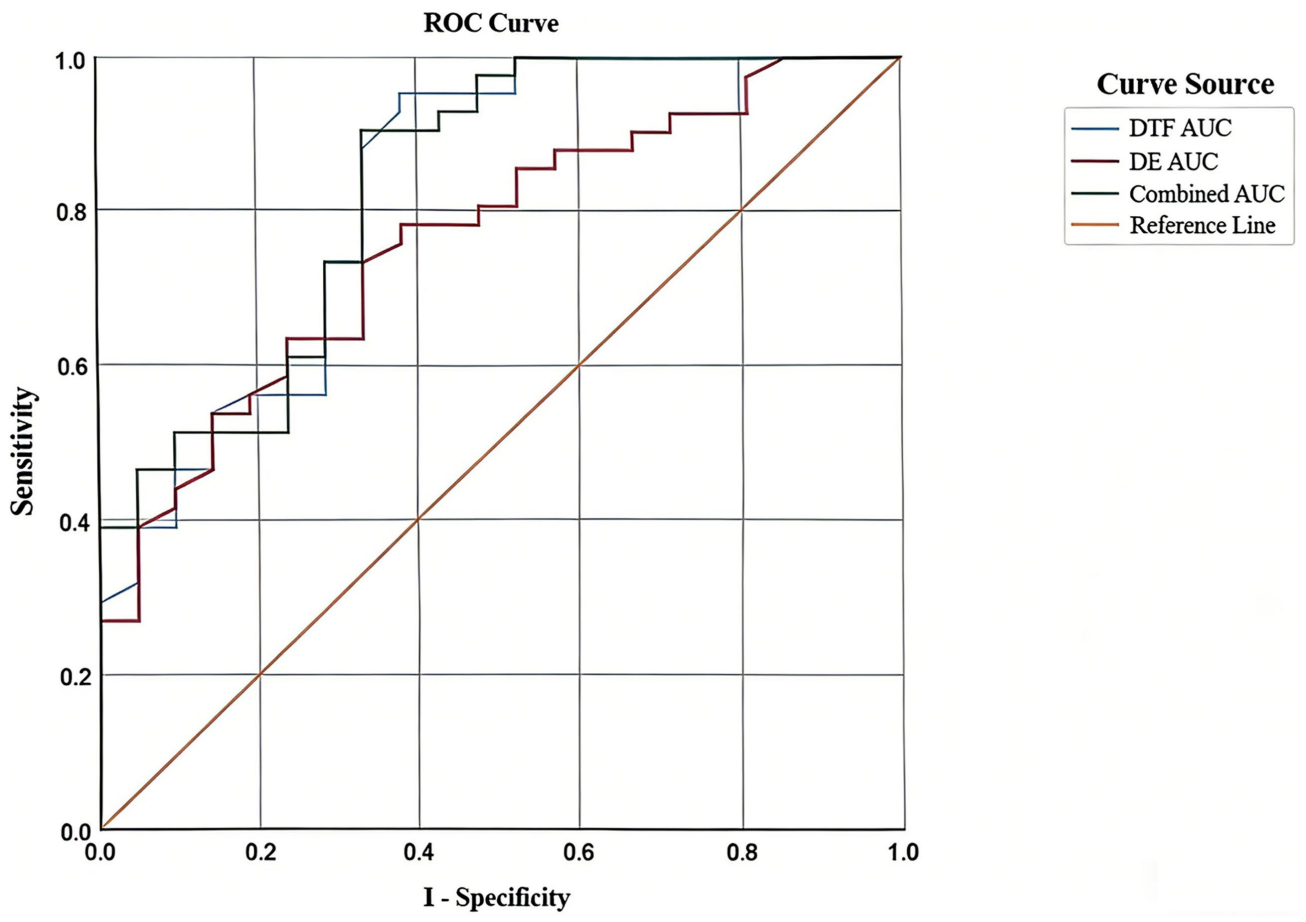
ROC curves of DE and DTF for predicting weaning failure.

In the control group, DE and DTF were used as test variables, with weaning failure as the state variable, to construct ROC curves. As shown in Figure 1, the AUC for DE was 0.760 (*p* < 0.01, 95% CI: 0.639–0.881), with a standard error of 0.062. The AUC for DTF was 0.825 (*p* < 0.01, 95% CI: 0.711–0.938), with a standard error of 0.058. The combined ROC curve of DE and DTF yielded an AUC of 0.830 (*p* < 0.01, 95% CI: 0.722–0.939), with a standard error of 0.056 (Figure 1).

### 3.4 Consistency between first SBT results and weaning outcomes

In the control group, among patients who passed SBT, 41 achieved successful weaning while 9 failed; among those who did not pass SBT, 12 did not achieve successful weaning. In the DTF group, among patients who passed SBT, 40 achieved successful weaning and 2 failed; among those who did not pass SBT, 22 did not achieve successful weaning. In the DTF + DE group, among patients who passed SBT, 40 achieved successful weaning and only 1 failed; among those who did not pass SBT, 20 did not achieve successful weaning.

Consistency analysis of first SBT results and final weaning outcomes is shown in Table 1. The Kappa value was 0.638 (*p* < 0.01) in the control group, 0.932 (*p* < 0.01) in the DTF group, and 0.963 (*p* < 0.01) in the DTF + DE group. All groups demonstrated strong consistency between first SBT results and weaning outcomes, with higher Kappa values in the DTF and DTF + DE groups compared with the control group (Table 3).

**Table 3:** Consistency analysis between initial SBT results and weaning outcomes in each group.

Group	SBT outcome	Weaning outcome		Kappa	*P*
		Success	Failure		
Control (*n* = 62)	Success	41 (66.13)	9 (14.52)	0.638	<0.01
	Failure	0 (0)	12 (19.35)		
DTF (*n* = 65)	Success	40 (61.54)	2 (3.08)	0.934	<0.01
	Failure	0 (0)	23 (35.38)		
DTF + DE (*n* = 61)	Success	40 (65.57)	1 (1.64)	0.963	<0.01
	Failure	0 (0)	20 (32.79)		

SBT, spontaneous breathing trial. Values are counts (*n*) of patients cross-classified by initial SBT outcome (success/failure) and subsequent weaning outcome (success/failure). Agreement between SBT results and weaning outcomes was evaluated using Cohen’s kappa (Kappa), with corresponding *P* values testing kappa against zero agreement.

## 4 Discussion

### 4.1 Discriminative performance and bedside feasibility of diaphragm ultrasound parameters

ROC analysis in this study showed that DTF achieved an AUC of 0.825 for predicting weaning failure, superior to DE (AUC = 0.760), while their combination yielded an AUC of 0.830 with limited incremental value. This finding suggests that DTF alone provides strong predictive efficacy, whereas DE, though valuable, offers only marginal additive benefit. From the perspective of bedside feasibility, a DTF-based stratification strategy is simpler, maintaining discriminative power while allowing rapid implementation by nurses in routine workflows.

Previous studies have mostly focused on single parameters. DiNino et al. first reported high sensitivity and specificity of DTF, with cutoffs commonly between 20 and 30% (9). The systematic review and meta-analysis conducted by Elfeky et al. further demonstrated that DTF achieved a higher overall AUC for predicting extubation success compared with traditional respiratory mechanics parameters (10). Using 20% as a cutoff in this study produced consistent results, supporting its applicability in nurse-led practice within neurosurgical ICUs. In contrast, DE showed lower predictive accuracy, likely influenced by imaging windows, probe angles, and patient body habitus, thus requiring greater operator experience. Jesus et al. reported lower reproducibility of DE compared with thickness measurements, aligning with the limited added value of DE observed here (11).

It is noteworthy that although DE alone is inferior to DTF, combined assessment may strengthen confidence in borderline cases. For instance, when DTF approaches 27%, DE ≥ 1.3 cm may indicate sufficient diaphragmatic motion to support extubation (12). Thus, despite limited statistical gain, DE may still serve as supplementary information in uncertain individual cases.

Physiologically, DTF reflects relative fiber thickening during diaphragmatic contraction, closely approximating endurance and workload, which explains its stable predictive performance (13). DE reflects displacement amplitude, influenced by chest wall compliance, lung volume, and inspiratory drive, making interpretation more complex in patients with impaired compliance or altered neural drive (14). This may explain why DE was less predictive than DTF in neurosurgical populations. Previous studies also suggested that DTF provides independent discrimination of weaning tolerance, whereas DE requires integration with other indices (15). Therefore, DTF is better suited as the primary stratification tool, while DE may serve as an adjunct in complex cases, especially for identifying insufficient drive or restricted motion.

### 4.2 Impact of diaphragm-based stratified SBT strategy on extubation stability

This study indicates that diaphragm-based stratification can shift weaning from maximizing early SBT “pass rates” to improving post-extubation stability. First SBT success was numerically highest in the control group, although the between-group difference was not statistically significant, consistent with a more permissive conventional 30 min SBT. In contrast, reintubation was markedly lower in both diaphragm-guided groups (<10%) than in controls (25.81%; *p* < 0.05), suggesting fewer delayed failures after an apparently successful SBT. Although first weaning success did not differ significantly, agreement between initial SBT assessment and subsequent weaning outcome improved (Kappa 0.638 in controls vs. 0.932 and 0.963 in the DTF and DTF + DE groups), supporting better decision consistency. Importantly, total mechanical ventilation duration was shorter in the diaphragm-guided groups, implying that preventing downstream setbacks (e.g., reintubation and repeated weaning attempts) outweighed any delays from selective prolonged observation.

A plausible mechanism is a reduction of false positives. Conventional SBT criteria mainly reflect short-term respiratory and hemodynamic tolerance and may not capture respiratory muscle endurance; therefore, some borderline patients can tolerate 30 min yet fail after extubation because of delayed fatigue, representing false-positive SBT results (7). Mezidi et al. similarly noted that short SBTs may overestimate weaning capacity, whereas longer observation can unmask fatigue that emerges later (16). In our protocol, prolonged observation was applied selectively to patients below diaphragm thresholds, rather than universally, aiming to identify delayed intolerance without imposing delays on low-risk patients.

Stratification may, however, increase false negatives: extubation-capable patients may show transient instability during prolonged observation and be deferred unnecessarily (17). This trade-off likely contributes to the absence of a significant increase in first weaning success. From a safety standpoint, the clinical penalty of false negatives is mainly delayed liberation, whereas false positives lead to extubation failure and reintubation. Consistent with this risk asymmetry, Cheng et al. reported that extubation failure carries a worse prognostic impact than moderate prolongation of ventilation (18). Finally, Mahmoodpoor et al. found DTF to outperform conventional respiratory mechanics in predicting weaning outcomes (19); our prospective, multicenter findings provide convergent evidence that a DTF-based, nurse-performed bedside assessment can support a targeted, safety-oriented weaning strategy.

### 4.3 Nursing role transformation and optimization of weaning management enabled by diaphragm ultrasound

In neurosurgical ICUs, the organization of weaning differs from general ICUs. Physicians are often occupied with rounds, surgery, and outpatient duties, limiting their bedside availability, while readiness assessment and SBT initiation primarily fall to nurses. Following sedation interruption, rapid changes in consciousness, hemodynamics, and respiration require immediate nursing judgment. Incorporating diaphragm ultrasound into routine nursing assessments allows simultaneous functional monitoring and SBT screening within daily workflow, fitting the neurosurgical ICU model and addressing the demand for precision weaning.

This integration shifts the nursing role. Traditionally, nurses focused on monitoring and execution, relying on vital signs and experience, with limited ability to quantify respiratory muscle function (20). This study showed that nurse-led DTF measurement achieved predictive AUC (0.825) comparable to physician-led reports, and Kappa analysis demonstrated significantly improved agreement between SBT and extubation (21). This indicates that with standardized training, nurses can provide objective evidence equivalent to physicians, shifting weaning management from “experience-based” to “data-driven.”

The value of nursing also lies in advancing readiness assessments and improving efficiency. By performing diaphragm assessment and SBT immediately after sedation interruption, nurses minimized delays from waiting for physician rounds. Although stratification lowered first SBT success, it reduced reintubation and shortened cumulative ventilation, reflecting that early nursing intervention improved both stability and safety.

The nurse–physician collaboration model also evolved. Nurses now provide objective diaphragm parameters rather than solely reporting vital signs, forming a “nurse-trigger, physician-confirm” workflow that streamlined decision-making and strengthened team trust. Hirzallah et al. emphasized that nurses should play an active rather than passive role in weaning, and the findings here support this positioning (22).

From a disciplinary perspective, diaphragm ultrasound as a skill independently mastered by nurses provides new technical advantages in critical care. Historically physician-led, this evaluation was shown here to be reliably performed by nurses, producing reproducible data with direct clinical relevance. Many studies have proposed dynamic diaphragm thickness as a reliable endurance marker (10, 23). This study further demonstrates that when performed by nurses, such assessment directly influenced extubation outcomes. This practice strengthens nursing authority in respiratory monitoring and weaning, highlighting the unique value of nursing within multidisciplinary collaboration.

## 5 Conclusion

Nurse-performed diaphragm ultrasound can be implemented as a bedside assessment to support physician-led weaning evaluation in neurosurgical ICUs. In this multicenter prospective cohort, DTF-based stratification was associated with reduced reintubation and shorter total ventilation duration, whereas first SBT success and first extubation success did not differ significantly between groups. Future studies should validate standardized training and thresholds and determine whether this strategy improves outcomes in randomized, contemporaneous comparisons.

### 5.1 Limitations

This study has limitations. Group allocation was time-period based rather than randomized, so temporal bias (including seasonal variation such as respiratory infection prevalence) cannot be excluded. The diaphragm-guided phases used an extended observation window for patients below ultrasound thresholds; therefore, some benefit may reflect longer observation itself rather than DTF/DE stratification alone. Sedative exposure was not quantified across groups; although assessments were initiated only after patients reached a comparable arousal level (RASS ≥ − 2), residual confounding may remain.

## Data Availability

The raw data supporting the conclusions of this article will be made available by the authors, without undue reservation.
