# Supplementary material for: Nurse-performed diaphragm ultrasound integrated with spontaneous breathing trial criteria for risk stratification of extubation outcomes in neurosurgical critically ill patients: a multicenter prospective cohort study
Source: Front Med (Lausanne). 2026 Feb 9;13:1732944. doi: 10.3389/fmed.2026.1732944 (PMC12926349; doi:10.3389/fmed.2026.1732944)
Supplement: Supplementary file 2 [file Supplementary_file_2.docx]

Supplementary Figure 2. Detailed weaning management algorithm for the Control, DTF, and DTF+DE groups
